# The NEDD4-binding protein N4BP1 degrades mRNA substrates through the coding sequence independent of nonsense-mediated decay

**DOI:** 10.1016/j.jbc.2024.107954

**Published:** 2024-11-02

**Authors:** Wen Zheng, Jinjing Guo, Shuyan Ma, Rong Sun, Yihua Song, Yuanmeng Chen, Renfang Mao, Yihui Fan

**Affiliations:** 1 Department of Pathogenic Biology, School of Medicine, Nantong University, Nantong, China; 2 Laboratory of Medical Science, School of Medicine, Nantong University, Nantong, China; 3 Department of Stomatology, Affiliated Hospital of Nantong University, Medical School of Nantong University, Nantong, China; 4 Department of Pathophysiology, School of Medicine, Nantong University, Nantong, China

**Keywords:** N4BP1, KH domain, NYN domain, nonsense-mediated mRNA decay, Fos-C, C-rich motif

## Abstract

3′UTRs are recognized for their role in regulating mRNA turnover while the turnover of a specific group of mRNAs mediated by coding sequences (CDSs) remains poorly understood. N4BP1 is a critical inflammatory regulator *in vivo* with a molecular mechanism that is not yet clearly defined. Our study reveals that N4BP1 efficiently degrades its mRNA targets *via* CDS rather than the 3′-UTR. This CDS-dependent mRNA turnover mechanism appears to be a general feature of N4BP1, as evidenced by testing multiple mRNA substrates, such as Fos-C, Fos-B, Jun-B, and C-X-C motif chemokine ligand 1. Detailed mapping of the motif identified a crucial 33-nt (289–322) sequence near the 5′-end of Fos-C-CDS, where the presence of polyC is necessary for N4BP1-mediated degradation. Functional studies involving domain deletion and point mutations showed that both the K homology and N4BP1, YacP-like nuclease domains are essential for N4BP1 to restrict mRNA substrates. The function of N4BP1 in mRNA turnover is not dependent on nonsense-mediated decay as it efficiently restricts mRNA substrates even in cells deficient in UPF1, UPF3A, and UPF3B. Additionally, the function of N4BP1 is not reliant on LUC7L3 despite its known association with this protein. Our findings suggest that N4BP1 acts as an endoribonuclease to degrade mRNA substrates primarily through CDSs containing a C-rich motif.

The level of mRNA is mainly controlled by transcription in the nucleus and mRNA turnover in the cytoplasm. The activation of transcription has been extensively studied but the control of mRNA turnover is less explored. The mRNA surveillance pathway is a general mechanism to control the quality of mRNA, but the mechanism that regulates the turnover of a specific group of mRNAs is largely unknown (1, 2). Specific control of a group of mRNAs for turnover is generally through multiple characterized motifs in the 3′-UTR (3, 4, 5). For example, tristetraprolin inhibits Ras-dependent tumor vascularization and inflammation by targeting AU-rich elements in the 3′UTR of vascular endothelial growth factor A and TNF-α, respectively (6, 7). Besides AU-rich elements, there are a variety of *cis*-acting elements in the 3′UTR (4). Unlike the 3′UTR, the mRNA coding sequences (CDSs) are generally considered to be non-regulatory; however, recent data reveal a potential role of CDSs in the regulation of mRNA turnover (8).

Large-scale analysis of binding sites from hundreds of RNA-binding proteins (BPs) reveals significant sites in CDSs, which suggests a regulatory role of CDS in mRNA turnover (9, 10). However, there are few examples of mRNA stability being regulated *via* the CDS. One example is that coding region determinant–BP binds to the coding region determinant of c-myc mRNA and protects c-myc mRNA from endonuclease cleavage (11, 12).

The mRNA turnover is precisely controlled and monitored through complex pathways (13, 14). Nonsense-mediated mRNA decay (NMD) is a well-studied pathway in mRNA surveillance (15, 16). Proper NMD activity is essential for normal development, and NMD abnormalities may contribute to neurodegenerative diseases, aging, and cancer (17, 18, 19, 20). Upstream frameshift 1 (UPF1), an ATP-dependent RNA helicase, is a central player in the NMD pathway (21). UPF1-guided NMD not only degrades abnormal mRNAs but also controls the turnover of a large number of normal mRNAs (16, 22). However, it is unclear whether UPF1-guided NMD is associated with CDS-mediated mRNA turnover.

N4BP1 is a Nedd4-BP that was identified by a yeast two-hybrid screen (23). However, its function in vivo remained largely unknown until several groups, including us, established KO mouse models. N4BP1 restricts the immune response through downregulation of cytokines, such as interleukin 6 and C-X-C motif chemokine ligand 1 (CXCL1), and its function is inactivated by caspase-8 upon engagement of toll like receptor 4 and toll like receptor 3 (24, 25). N4BP1 is highly expressed in skin and restricts skin inflammation by blocking keratinocyte proliferation and neutrophil infiltration through the regulation of AP-1 transcriptional factors (26). N4BP1 has five identified domains, including two RNA-binding KH domains in the N-terminal, an N4BP1, YacP-like nuclease (NYN) ribonuclease domain in the C terminal, and two Ub-binding domains (UBA and CoCUN). N4BP1 has been suggested to be an endoribonuclease that degrades viral RNA, but how it recognizes and degrades cellular mRNAs are unknown (27).

The heterogeneous nuclear ribonucleoprotein K homology (hnRNP-KH) domain was first identified in the hnRNP-K about 28 years ago (28). The KH motif consists of approximately 70 amino acids and the binding cleft of one KH domain accommodates only four nucleic acid bases (29, 30). The typical function of KH domains is recognition of an ssRNA or ssDNA *via* diverse motifs. For example, the KH domains of hnRNP-K and poly (C) binding proteins bind cytosine-rich RNA, while KH domains from NuSA and Nova bind adenine-rich regions (31, 32, 33). N4BP1 has one KH-like domain and one KH domain in its N-terminal, but their role and recognized motif are unknown. In addition, N4BP1 contains an NYN domain, which is novel domain recognized by sensitive sequence searches recently (34). NYN domain was supposed to have ribonuclease activity because it shares a common protein fold with previously characterized nucleases domains including PilT N-terminal and FLAP. However, the role of NYN domain in N4BP1 and how it degrades mRNA targets are largely unexplored. Here, we found N4BP1 restricts mRNAs *via* CDSs and the motif in Fos-C mRNA locates at 200-nt region. The function of N4BP1 is dependent on its KH and NYN domain. Thus, our findings provided new insights to understand how CDSs control mRNA stability *via* N4BP1.

## Results

### Fos-C and Fos-B are targets of N4BP1

**Figure 1:**
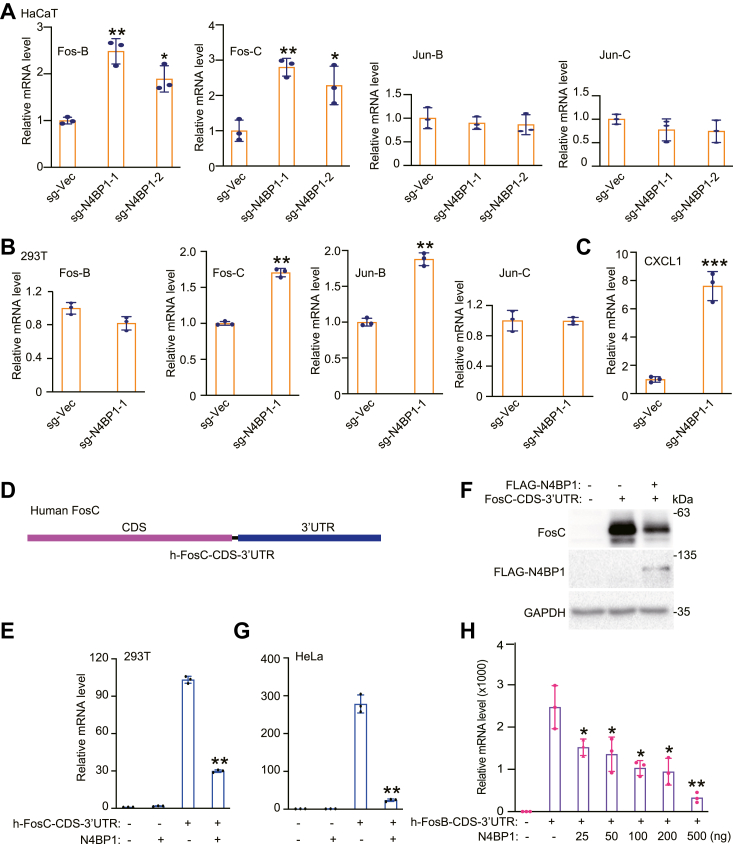
**Identification N4BP1 mRNA targets.***A*, RT-PCR analysis of the mRNA levels of Fos-B, Fos-C, Jun-B, and Jun-C in WT and N4BP1^−/−^ HaCaT cells. *B*, RT-PCR analysis of the mRNA levels of Fos-B, Fos-C, Jun-B, and Jun-C in WT and N4BP1^−/−^ 293T cells. *C*, RT-PCR analysis of the mRNA level of CXCL1 in WT and N4BP1^−/−^ 293T cells. *D*, schematic diagram of the mRNA structure of human Fos-C coding sequences and 3′-UTRs. *E*, RT-PCR analysis of the Fos-C level in 293T cells transfected with h-FosC-CDS-3′ UTR, f-prk-N4BP1 or their combination. *F*, Western blot analysis of Fos-C expression in 293T cells transfected with f-prk-N4BP1, h-FOSC-CDS-3′ UTR, or their combination. *G*, RT-PCR analysis of the Fos-C level in HeLa cells transfected with h-FosC-CDS-3′ UTR, f-prk-N4BP1, or their combination. *H*, RT-PCR analysis of Fos-B in 293T cells transfected with h-FosB-CDS-3′ UTR and different amount of f-prk-N4BP1. Data in (*A* and *B*) are representative of three independent experiments. Data in (*E–H*) are representative of four independent experiments. RT-PCR, n = 3, ∗*p* < 0.05; ∗∗*p* < 0.01; and ∗∗∗*p* < 0.001. CDS, coding sequence; CXCL1, C-X-C motif chemokine ligand 1; RT-PCR, reverse transcription-polymerase chain reaction.

Previously, we identified potential N4BP1 targets, including members of the AP-1 family and CXCL1, in mouse models. To explore the molecular mechanism of how N4BP1 recognizes and degrades its mRNA substrates, we confirmed these targets in HaCaT cells. Knocking down N4BP1 significantly upregulated the mRNA levels of Fos-C and Fos-B (Fig. 1*A*). Similarly, knocking down N4BP1 in 293T cells resulted in the upregulation of Fos-C, Jun-B, and CXCL1 (Fig. 1, *B* and *C*). Collectively, our results indicate that members of the AP-1 family are mRNA targets of N4BP1. But the targets in different cells might be slightly different in distinguished cells. Next, we cloned the CDS and 3′-UTR of human Fos-C into an expression vector (Fig. 1*D*). Overexpression of N4BP1 significantly inhibited Fos-C transcript in 293T cells (Fig. 1*E*). Western blotting confirmed the inhibitory effect of N4BP1 on Fos-C expression (Fig. 1*F*). Similar results were obtained in HeLa cells (Fig. 1*G*). N4BP1 also inhibited the CDS-3′UTR–generated human Fos-B transcript in a dose-dependent manner (Fig. 1*H*). Together, our results demonstrate that N4BP1 dramatically degrades its targeted mRNAs.

### N4BP1 degrades mRNA *via* CDSs

**Figure 2:**
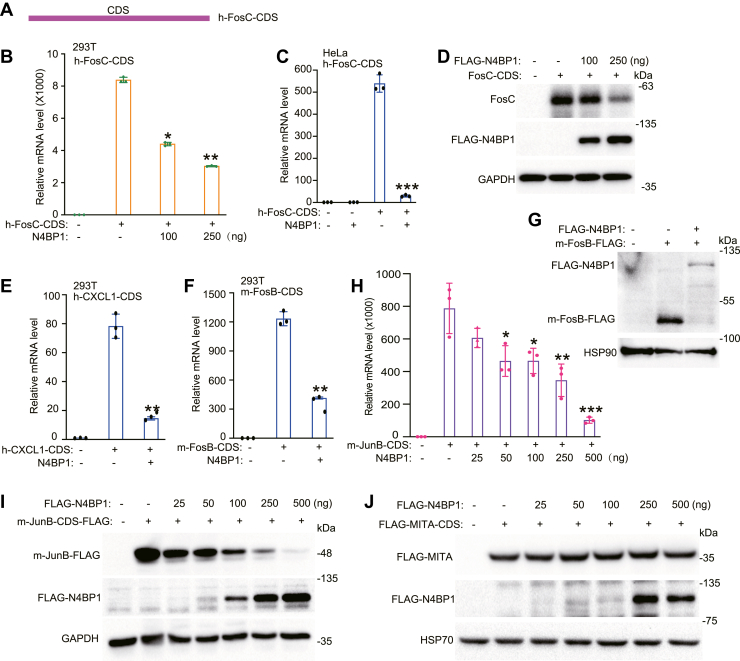
**N4BP1 restricts mRNA *via* coding sequences.***A*, schematic diagram of the structure of human Fos-C coding sequences (h-Fos-C-CDS). *B*, 293T cells were transfected with h-Fos-C-CDS and different amount of f-prk-N4BP1 for RT-PCR analysis. *C*, HeLa cells were transfected with h-Fos-C-CDS and f-prk-N4BP1 for RT-PCR analysis. *D*, 293T cells were transfected with h-Fos-C-CDS and different amount of f-prk-N4BP1 for WB analysis. *E*, 293T was transfected with h-CXCL1-CDS and f-prk-N4BP1, and the level of Fos-C was detected by RT-PCR. *F*, 293T cells transfected with m-Fos-B-CDS and f-prk-N4BP1 were used for RT-PCR analysis of m-Fos-B. *G*, 293T cells transfected with m-Fos-B-CDS and f-prk-N4BP1 were used for Western blot analysis of Flag-Fos-B and Flag-N4BP1. *H*, 293T cells were transfected with m-Jun-B-CDS and different amount of f-prk-N4BP1 for RT-PCR analysis of m-Jun-B. *I*, 293T cells were transfected with m-Jun-B-CDS and different amount of f-prk-N4BP1 for WB analysis of m-Jun-B. *J*, 293T cells were transfected with MITA-CDS and different amount of f-prk-N4BP1 for WB analysis of FLAG-MITA. Data in (*B–I*) are representative of three independent experiments. Data in (*J*) are representative of two independent experiments. RT-PCR, n = 3, ∗*p* < 0.05; ∗∗*p* < 0.01; and ∗∗∗*p* < 0.001. CDS, coding sequence; CXCL1, C-X-C motif chemokine ligand 1; RT-PCR, reverse transcription-polymerase chain reaction.

In general, the 3′UTR is critical for mRNA turnover. To identify the motif recognized by N4BP1, we deleted the 3′UTR of human Fos-C (Fig. 2*A*). Ectopic expression of N4BP1 still decreased the levels of the CDS-generated Fos-C transcript in 293T cells (Fig. 2*B*); similar results were obtained in HeLa cells (Fig. 2*C*). Western blotting confirmed that N4BP1 inhibits Fos-C protein production in a dose-dependent manner (Fig. 2*D*). Our results indicate that N4BP1 inhibits mRNA targets *via* the CDS. To examine whether N4BP1-mediated degradation of mRNA *via* CDS is a general mechanism or is target-specific, we used another N4BP1 target, CXCL1, and showed that N4BP1 inhibited CDS-generated human CXCL1 (Fig. 2*E*). Next, we explored whether N4BP1-mediated mRNA degradation is conserved between human and mouse. Ectopic expression of N4BP1 also inhibited CDS-generated mouse Fos-B at the mRNA and protein levels (Fig. 2, *F* and *G*). Similarly, N4BP1 inhibited mouse-CDS–encoded Jun-B mRNA *via* the CDS (Fig. 2, *H* and *I*). The inhibitory effect of N4BP1 on Fos-C, Fos-B, Jun-B, and CXCL1 was specific because N4BP1 did not inhibit expression of MITA (mediator of IRF3 activation, also known as STING) when coexpressed (Fig. 2*J*). Together, our results indicate that N4BP1 restricts specific mRNA targets *via* the CDS.

### Precise mapping of the essential RNA sequence in the CDS of Fos-C

**Figure 3:**
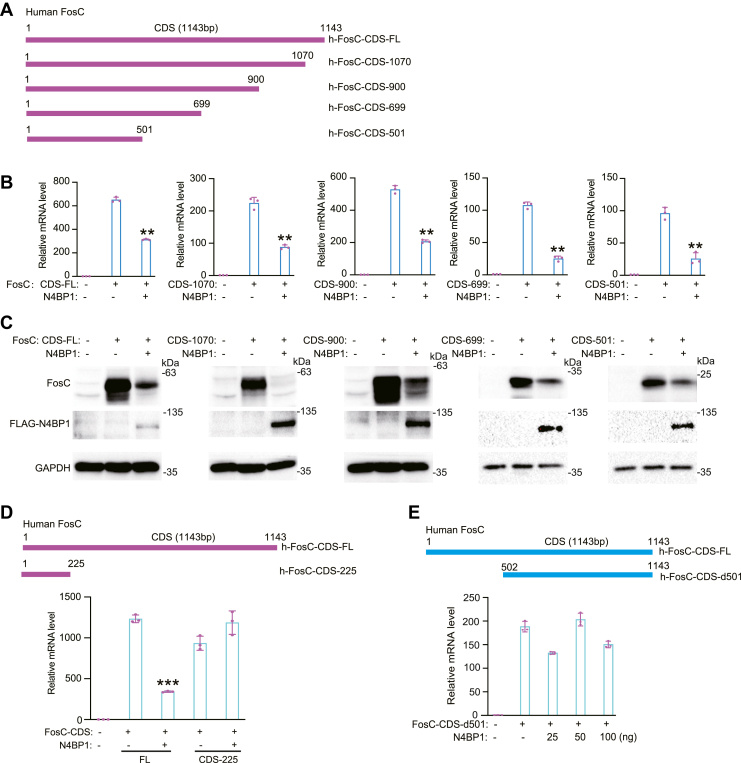
**Mapping the mRNA motif in Fos-C-CDS.***A*, schematic diagram demonstrates the full length of human Fos-C-CDS and different mutants with deleted 3′-end. *B*, 293T was transfected with CDS-FL, CDS-1070, CDS-900, CDS-699, CDS-501 and f-PRK-N4BP1, and the level of Fos-C was detected by RT-PCR. *C*, detection of Fos-C by Western blot in 293T was transfected with CDS-FL, CDS-1070, CDS-900, CDS-699, CDS-501, and f-PRK-N4BP1. *D*, *top*: schematic indication of h-Fos-C-CDS-FL and h-FosC-CDS-225; *below*: 293T was transfected with 100 ng of CDS-FL, CDS-225, and 50 ng of f-prk-N4BP1, and the level of Fos-C was detected by RT-PCR. *E*, *top*: schematic demonstration of deletion of Fos-C-CDS at 5′-end. *Bottom*: 293T cells were transfected with 1 ug of FosC-CDS-d501 and 100 ng of f-prk-N4BP1 or 25 ng, 50 ng of f-prk-N4BP1 plus desired vector for a total of 100 ng of DNA. RT-PCR was used to detect Fos-C. Data in (*B–E*) are representative of three independent experiments. RT-PCR, n = 3, ∗*p* < 0.05; ∗∗*p* < 0.01; and ∗∗∗*p* < 0.001. CDS, coding sequence; RT-PCR, reverse transcription-polymerase chain reaction.

To identify the essential RNA sequence in Fos-C-CDS for N4BP1-mediated restriction, we made a series of deletions in the 3′-end (1070–1143, 900–1143, 699–1143, and 501–1143) (Fig. 3*A*). RT-PCR was used to examine the effect of N4BP1 on these sequences. N4BP1 degrades all transcripts with a series of 3′-deletions (Fig. 3*B*). These data indicate that the essential RNA sequence is located within the 500 nt of the 5′-end. Western blotting confirmed the inhibitory effect of N4BP1 on all 3′-deletions (Fig. 3*C*). Further deletion indicated that N4BP1 failed to degrade transcript from the 5′-225-nt fragment of Fos-C-CDS (Fig. 3*D*). These results suggest an essential role of the 275 nt between 225 and 500 nt in Fos-C-CDS. Next, we deleted the 501 nt from the 5′ region and N4BP1 no longer inhibited the Fos-C mRNA transcript (Fig. 3*E*). Together, our results support the essential role of the 275-nt sequence between 225 and 500 nt of the Fos-C-CDS mRNA.

### N4BP1 degrades Fos-C *via* polyC regions in the CDS

**Figure 4:**
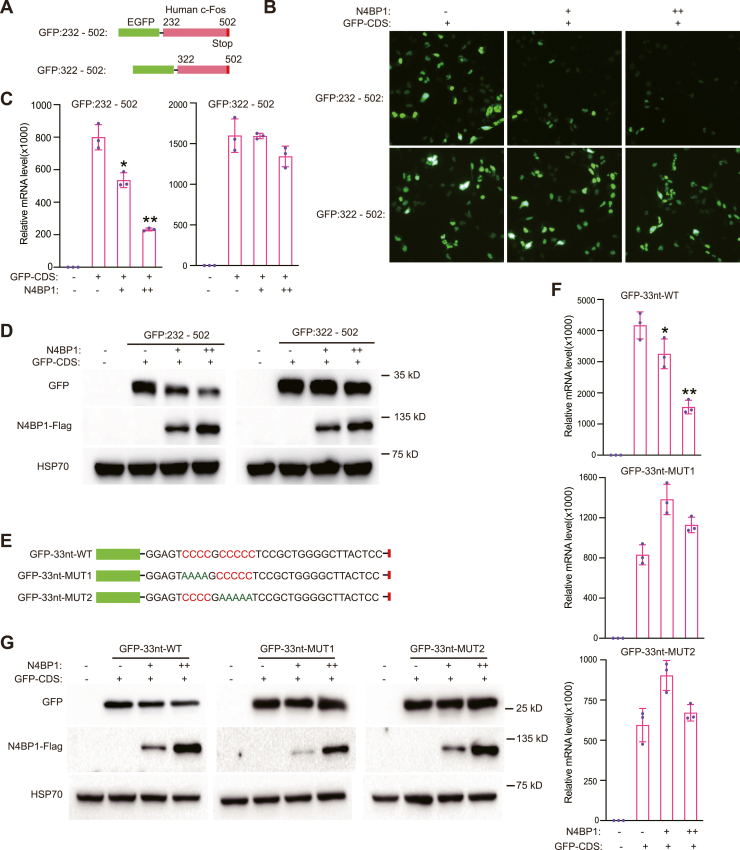
**A polyC containing 33-nt region from Fos-C is sufficient to promote N4BP1-mediated targets degradation.***A*, schematic diagram showing the EGFP fusion plasmid of h-FosC-CDS (232–502) and h-FosC-CDS (322–502). *B*, the fluorescence intensity was observed by photographing under a fluorescence microscope with a 10× field of view. *C*, RT-PCR was used to detect the mRNA level of GFP with different fusion. The plasmids were transfected as indicated. *D*, Western blot analysis of GFP and N4BP1 expression in cells that transfected with plasmids encoding GFP with different fusion. *E*, schematic showing EGFP fusion core sequences and C-rich mutants. *E*, 293T cells were transfected with N4BP1 and GFP-33nt-WT or mutants, and the mRNA level of GFP were detected by RT-PCR. *E*, 293T cells were transfected with N4BP1 and GFP-33nt-WT or mutants, and the expressions of N4BP1 and GFP were detected by Western blot. Data in *B–D*, *F*, and *G* are representative of three independent experiments. RT-PCR, n = 3, ∗*p* < 0.05; ∗∗*p* < 0.01; and ∗∗∗*p* < 0.001. CDS, coding sequence; RT-PCR, reverse transcription-polymerase chain reaction.

To explore the mRNA features recognized by N4BP1, we examined whether the 275-nt RNA region is sufficient for N4BP1 degradation. We fused the 232-502-nt region to GFP and also made a GFP fusion with a deletion containing 322 to 502 nt (Fig. 4*A*). The 232 to 502 region promoted N4BP1-mediated GFP degradation (Fig. 4*B*), but the 322 to 502 region did not promote N4BP1-mediated GFP degradation (Fig. 4*B*). This result indicates that the 232 to 322 region is important for N4BP1-mediated degradation of mRNA targets. To confirm this finding, we also examined the mRNA level of GFP that fused with 232 to 502 or 322 to 502 nt upon N4BP1 overexpression (Fig. 4*C*). N4BP1 significantly degrades GFP transcript that fused with 232 to 502 but not 322 to 502 nt (Fig. 4*C*). Western blotting demonstrated that N4BP1 inhibited GFP production when it was fused with the 232 to 502 fragment, but not when it was fused with the 322 to 502 fragment (Fig. 4*D*). In the 232 to 322 regions, there is a C-rich domain (Fig. 4*E*). Thus, we cloned a 33-nt region from 289 to 322, which includes the C-rich domain, and fused it to GFP (Fig. 4*E*). We also replaced the polyC region with polyA (Fig. 4*E*). The GFP-fused polyC region from Fos-C was sufficient for N4BP1-mediated degradation of GFP transcript (Fig. 4*F*). However, the GFP transcripts fused with polyA mutants were not degraded by N4BP1 (Fig. 4*F*). At protein level, we got similar results that N4BP1 inhibits GFP production when GFP is fused with polyC but not polyA mutants (Fig. 4*G*). Together, our results show that the polyC region in the 289 to 322 (33 nt) regions from FosC-CDS is required for N4BP1-mediated mRNA degradation.

### The KH domain is required for N4BP1 restriction function

**Figure 5:**
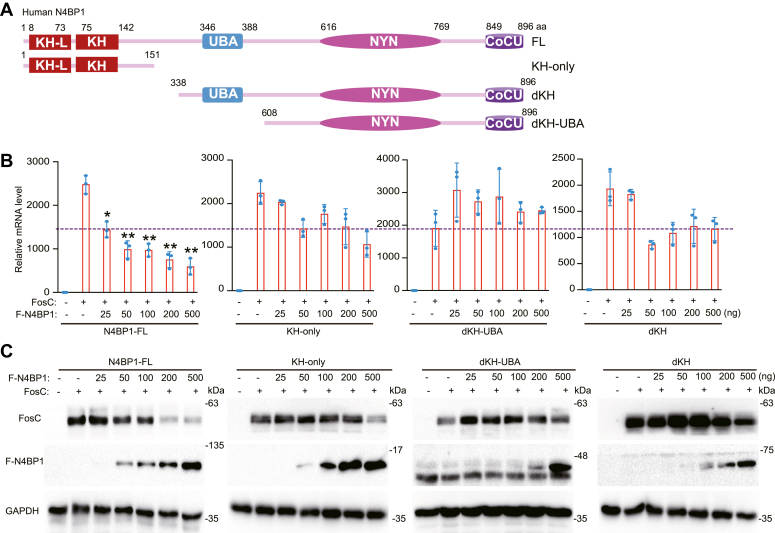
**N4BP1 degrades mRNA targets dependent on KH domain.***A*, schematic diagram demonstrates full-length N4BP1 or N4BP1 deletion mutants with FLAG tags at their N-terminal. *B*, 293T was transfected with different amount of N4BP1-FL, KH-only, dKH-UBA, dKH, along with Fos-C, and then the Fos-C was detected by RT-PCR. *C*, 293T was transfected with different amount of N4BP1-FL, KH-only, dKH-UBA, dKH, along with Fos-C, and then the Fos-C was detected by WB. Data in (*B* and *C*) are representative of three independent experiments. RT-PCR, n = 3, ∗*p* < 0.05; ∗∗*p* < 0.01; ∗∗∗*p* < 0.001. CDS, coding sequence; KH, K homology; RT-PCR, reverse transcription-polymerase chain reaction.

N4BP1 has one KH-like domain and one KH domain in its N-terminal. The KH domain is known to bind with mRNA, but its role in N4BP1 is unclear. To explore the function of the KH domain, we made truncation mutants of N4BP1, including KH-only, dKH, and dKH-UBA (Fig. 5*A*). Full-length N4BP1 dose dependently decreased levels of the Fos-C transcript but the dKH and dKH-UBA mutants did not (Fig. 5*B*). However, the KH-domain–only mutant (KH-only) is not sufficient to perform the inhibitory effect (Fig. 5*B*). By Western blotting, full-length N4BP1 inhibited Fos-C in a dose-dependent manner but all three mutants (KH-only, dKH, and dKH-UBA) did not (Fig. 5*C*). Thus, our results indicate that the KH domain is required for N4BP1’s inhibitory function but the KH domain alone is not sufficient.

### The RNA-binding activity of the KH domain and the endoribonuclease activity of the NYN domain are required for N4BP1 function

**Figure 6:**
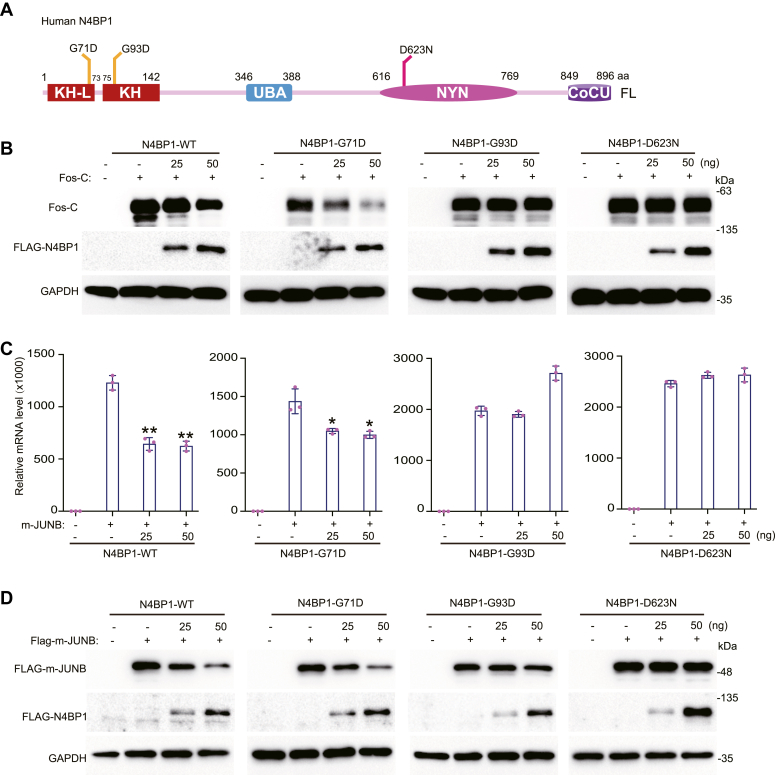
**N4BP1 degrades mRNA targets dependent on KH and NYN domain.***A*, schematic diagram of point mutations in the KH and NYN domains of N4BP1. *B*, 293T was transfected with N4BP1-WT, N4BP1-G71D, N4BP1-G93D, N4BP1-D623N, and Fos-C, and then Fos-C and Flag-N4BP1 were analyzed by Western blot. *C*, 293T was transfected with N4BP1-WT, N4BP1-G71D, N4BP1-G93D, N4BP1-D623N and flag-m-JUNB, and then the mRNA level of m-JUNB was detected by RT-PCR. *D*, 293T was transfected with N4BP1-WT, N4BP1-G71D, N4BP1-G93D, N4BP1-D623N, and flag-m-JUNB, and then m-JUNB was detected by Western blot. Data in (*B–D*) are representative of three independent experiments. RT-PCR, n = 3, ∗*p* < 0.05; ∗∗*p* < 0.01; and ∗∗∗*p* < 0.001. KH, K homology; NYN, N4BP1, YacP-like nuclease; RT-PCR, reverse transcription-polymerase chain reaction.

The binding of the KH domain relies on its conserved GXXG amino acid. We produced G71D and G93D mutants to disrupt the mRNA-binding activity of the KH-like and KH domains, respectively (Fig. 6*A*) (35). We also produced a D623N mutant to disrupt the endoribonuclease activity (27). The G71D mutant efficiently inhibited Fos-C and its action was comparable to WT N4BP1; however, the G93D and D623N mutants lost their inhibitory function (Fig. 6*B*). Therefore, the RNA-binding activity of the KH domain and the endoribonuclease activity of N4BP1 are required for N4BP1 function. To support this conclusion, we used another N4BP1 target, Jun-B. Overexpression of WT N4BP1 significantly decreased the level of the Jun-B transcript, as did the G71D mutant (Fig. 6*C*). However, the G93D and D623N mutants lost their inhibitory effect on the Jun-B transcript (Fig. 6*C*). Western blotting showed similar results (Fig. 6*D*). Together, our results suggest the requirement of mRNA-binding activity of the KH domain and the endoribonuclease activity of the NYN domain for proper N4BP1 function.

### The function of N4BP1 is independent of NMD

**Figure 7:**
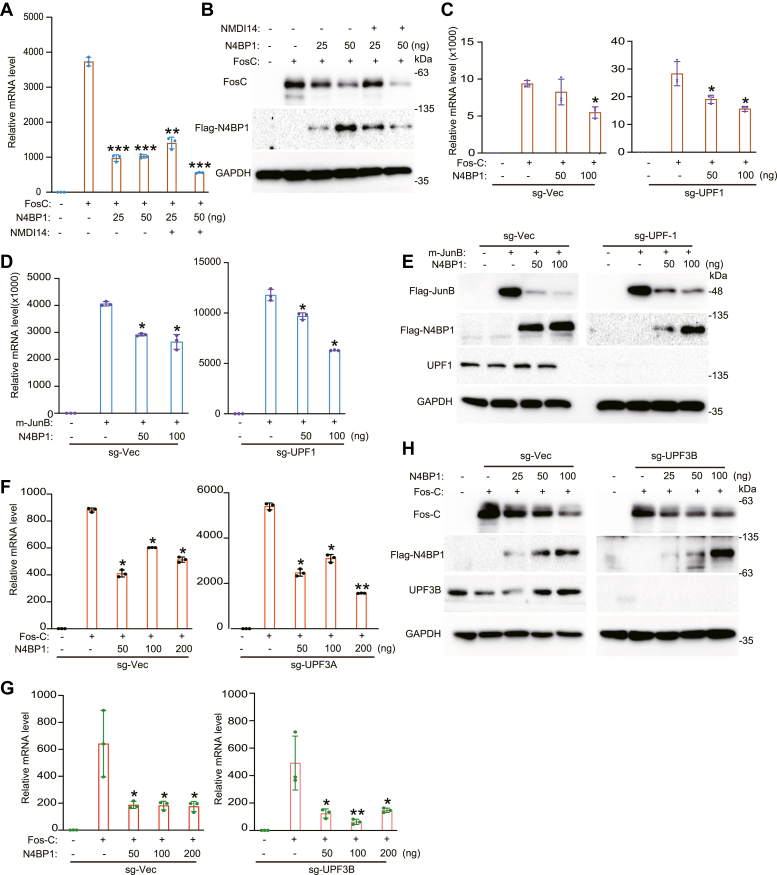
**N4BP1 restricts mRNA is independent on nonsense-mediated mRNA decay.***A*, 293T was transfected with f-prk-N4BP1 and Fos-C. After 8 h, the medium was replaced with a fresh medium along with or without 5 μM of NMDI14. Cells were harvested after 48 h. Fos-C was detected by RT-PCR. *B*, 293T was transfected with f-prk-N4BP1 and Fos-C. After 8 h, the medium was replaced with a fresh medium along with or without 5 mM of NMDI14. Cells were harvested after 48 h. Fos-C was detected by WB. *C*, f-prk-N4BP1 and Fos-C were transfected in sg-Vec cell line and sg-UPF1 cell line, respectively. The level of Fos-C was detected by RT-PCR. *D*, f-prk-N4BP1 and Jun-B were transfected in sg-Vec cell line and sg-UPF1 cell line, respectively. The level of Jun-B was detected by RT-PCR. *E*, f-prk-N4BP1 and Jun-B were transfected in sg-Vec cell line and sg-UPF1 cell line, respectively. The level of Jun-B was detected by WB. *F*, f-prk-N4BP1 and Fos-C were transfected in sg-Vec cell line and sg-UPF3A cell line, respectively. The level of Fos-C was detected by RT-PCR. *G*, f-prk-N4BP1 and Fos-C were transfected in sg-Vec cell line and sg-UPF3B cell line, respectively. The level of Fos-C was detected by RT-PCR. *H*, f-prk-N4BP1 and Fos-C were transfected in sg-Vec cell line and sg-UPF3B cell line, respectively. The level of Fos-C was detected by WB. Data in (*A* and *B*) are representative of two independent experiments. Data in (*C–H*) are representative of three independent experiments. RT-PCR, n = 3, ∗*p* < 0.05; ∗∗*p* < 0.01; and ∗∗∗*p* < 0.001. RT-PCR, reverse transcription-polymerase chain reaction; UPF1, upstream frameshift 1.

To examine whether N4BP1-mediated mRNA restriction is dependent on NMD, we treated cells with NMDI14, a potent NMD inhibitor. In the presence of NMDI14, overexpression of N4BP1 decreased the levels of the Fos-C transcript (Fig. 7*A*). Western blotting showed similar results (Fig. 7*B*). These results suggest that N4BP1 restricts mRNA substrates independently of NMD. To further explore the role of NMD in the function of N4BP1, we made UPF1 KO cells. N4BP1 efficiently decreased the levels of the Fos-C transcript in UPF1-deficient cells (Fig. 7*C*). N4BP1 strongly decreased the levels of the Jun-B transcript in control cells and UPF1-deficient cells (Fig. 7, *D* and *E*). Because UPF1 is a helicase with essential roles in NMD, these results support that N4BP1 function does not rely on NMD. Because UPF3A and UPF3B are also involved in NMD, we examined whether the function of N4BP1 is dependent on these two molecules. N4BP1 decreased the levels of the Fos-C transcript in UPF3A- or UPF3B-deficient cells (Fig. 7, *F* and *G*). Western blotting confirmed that N4BP1 inhibits the expression of Fos-C in UPF3B-deficient cells (Fig. 7*H*). Collectively, our results demonstrate that N4BP1 restricts mRNA substrates independently of NMD and is also independent of UPF1, UPF3A, and UPF3B.

### The function of N4BP1 is independent of LUC7L3

**Figure 8:**
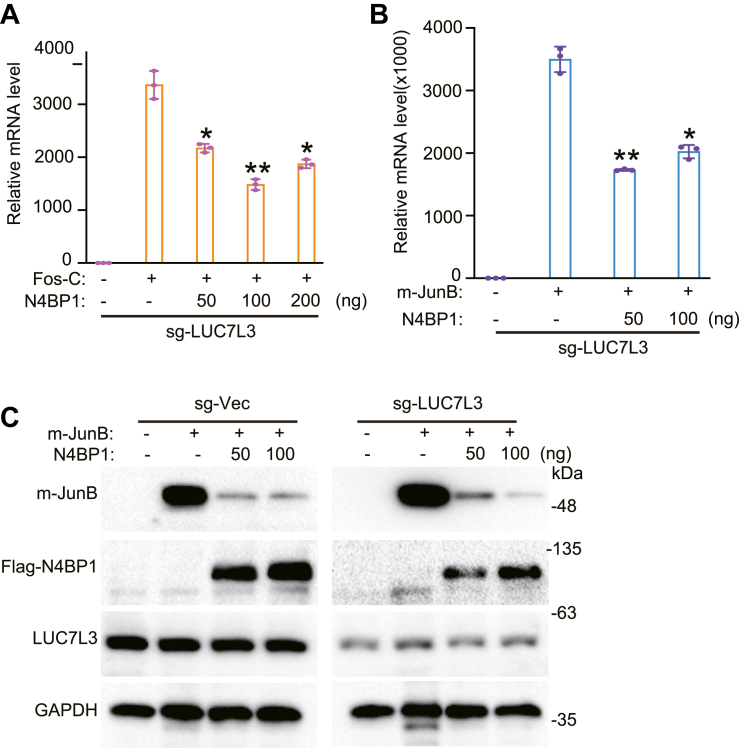
**N4BP1 restricts mRNA is independent of LUC7L3.***A*, sg-LUC7L3 cell line was transfected with f-prk-N4BP1 and Fos-C, and Fos-C was detected by RT-PCR. *B*, sg-LUC7L3 cell line was transfected with f-prk-N4BP1 and Flag-m-Jun-B, and m-JUNB was detected by RT-PCR. *C*, control and sg-LUC7L3 cells were transfected with f-prk-N4BP1 and Jun-B, and the level of Jun-B was detected by WB. The control sample of sg-Vec is the same with it used in Figure 7*E*. The FLAG-N4BP1 panel of sg-Vec in Figure 7*E* was reused at here. Data in (*A–C*) are representative of three independent experiments. RT-PCR, n = 3, ∗*p* < 0.05; ∗∗*p* < 0.01; and ∗∗∗*p* < 0.001. RT-PCR, reverse transcription-polymerase chain reaction.

In a previous publication, by mapping the N4BP1-associated proteins using mass spectrometry, LUC7L3 was associated with N4BP1 in neuroblastoma cells (36) (original data obtained from Professor Boes). Thus, we established LUC7L3-deficient cells and found that N4BP1 decreased the level of Fos-C transcript in a dose-dependent manner in LUC7L3-deficient cells (Fig. 8*A*). Using another N4BP1 substrate, N4BP1 also powerfully inhibits the transcript of Jun-B in dose-dependent manner (Fig. 8*B*). Western blot further confirmed that N4BP1 inhibits the expression of Jun-B in LUC7L3-deficient cells as it did in WT cells (Fig. 8*C*). Taken together, our results demonstrate that the function of N4BP1 is independent on LUC7L3.

## Discussion

Genetic disruption of the N4BP1 gene in mice revealed a critical role of N4BP1 in the immune response and the regulation of cytokines; however, the molecular mechanism of N4BP1 activity is largely unknown (24, 25, 26). N4BP1 is proposed to be an endoribonuclease but how it recognizes and degrades mRNA substrates is unclear. Here, we show that N4BP1 efficiently restricts transcripts of multiple mRNA substrates such as Fos-C, Fos-B, Jun-B, and CXCL1. N4BP1 restriction of mRNA substrates is dependent on its KH and NYN domains. Unlike regnase-1, the function of N4BP1 is independent of UPF1 and NMD (37, 38). Most importantly, we found that N4BP1 restricts mRNA substrates *via* their CDSs. Although CDS-mediated mRNA decay has been investigated and coding region mRNA stability determinants have been mapped in several mRNAs, such as C-Myc and Fos-C, the critical proteins involved in this process are largely unknown (11, 39, 40). Here, we found that N4BP1 restricts mRNA substrates through the CDS, which suggests that N4BP1 is a regulator of CDS-regulated mRNA decay.

For the Fos-C mRNA substrates, we precisely mapped the RNA sequence that is essential for N4BP1 function. We narrowed the RNA sequences into 33-nt (289–322) region near 5′-end of CDS. This region is enriched with C and the polyC is required for N4BP1-mediated targets degradation. Interestingly, downstream this region includes a previously identified 87-nt minimal destabilizing element (41). The 87-nt minimal destabilizing element was shown to associated with a protein complex including five proteins that are UNR, a purine-rich RNA BP; PABP, a poly(A) BP; PAIP-1, a poly(A) BP interacting protein; hnRNP D, an AU-rich element BP; and NSAP1, an hnRNP R–like protein (41). However, the relationship and their discrepancy between the 33 nt and 87 nt are unknown. It is also interesting to test whether N4BP1 is associated with this protein complex and their functional relationship.

The KH domain is approximately 70 amino acids long and has a conserved consensus sequence VIGXXGXXI in the middle of the domain (29, 30, 42). The typical function of KH domains is to recognize an ssRNA or ssDNA. Based on the conserved domain database of NCBI, N4BP1 has two KH domains in its N-terminal, one is a KH-like domain and another one is a KH domain (www.ncbi.nlm.nih.gov/Structure/cdd/cdd.shtml) (43). The KH domain has an RNA-binding GXXG motif, and the glycine at position 93 (G93) is a potential RNA-binding site. The KH-like domain is a divergent KH domain that lacks the RNA-binding GXXG motif. To determine the role of the RNA-binding activity of N4BP1, we made two mutants, G93D and G71D, to disrupt the RNA-binding activity of the KH domain and the KH-like domain, respectively. The G93D mutant completely abolished the inhibitory function of N4BP1, while the G71D mutant retained the function. Our results indicate that N4BP1 relies on its RNA-binding activity to recognize mRNA substrates, but the role of the KH-like domain in RNA recognition needs to be further explored.

In summary, we provide evidence that N4BP1 is an endoribonuclease that restricts mRNA substrates. N4BP1 has RNA-binding and ribonuclease activity, both of which are required for N4BP1 function. Notably, N4BP1 restricts mRNA substrates *via* their CDSs. Our findings demonstrate that N4BP1 might be a critical regulator in CDS-mediated mRNA decay.

## Experimental procedures

### Cell culture and treatment

HEK293T, HeLa, and HaCaT were cultured in Dulbecco's modified Eagle's medium containing 10% fetal bovine serum and 1% penicillin-streptomycin antibiotic mixture, which required a culture environment of 37 °C and 5% of CO_2_. To obtain N4BP1^−/−^ HEK293T or N4BP1^−/−^ HaCaT cells, we designed three pairs of sgRNAs with the following sequences (Table S2). EpiCRISPR vectors have been described previously (44). The targeted single guide RNA was inserted into the vector by digestion and ligation, and then verified by DNA sequencing. The obtained KO plasmid was transfected at a ratio of 1:1 using Lipofectamine 2000 (Invitrogen), and the epiCRISPR vector plasmid was used as a control. Forty-eight hours after transfection, puromycin was used for treatment until stable cell lines were established and verified by Western blotting. Likewise, the sgUPF1, sgUPF3B, sgUPF3A, and sgLUC7L3 cell lines used in the experiments were obtained in this way.

### Plasmids and transient transfection

H-OE-pEGFP-CXCL1, m-OE-pECMV-Fos-B-Flag, and m-OE-pECMV-Jun-B-Flag were ordered from http://www.miaolingbio.com. H-OE-pcDNA3.1(−)-Fos-C CDS, h-OE-pcDNA3.1(−)-Fos-C CDS+3′UTR, h-OE-Fos-B CDS, h-OE-Fos-B CDS+3′UTR were amplified by using high-fidelity thermostable DNA polymerase (2 × Phanta Max Master Mix) with the designed specific primers, and the resulting PCR products were digested with EcoR1 and BamH1, and then ligated into pCDH-Flag or pcDNA3.1 vector. Different truncations of h-Fos-C-CDS were made refer to the above method. The primers are provided in the Table S3. For various plasmids with Flag-Tag, the FLAG CDS GATTACAAGGACGACGATGACAAG was fused with target gene sequence, and then the gene fragment was cloned into the PRK vector. The conventionally cultured cells were seeded in a 6-well plate, and 2 ml of complete medium was added, mixed well, and placed in a carbon dioxide incubator at 37 °C overnight. Cell density up to about 70% can be used for experiments. Cells were transfected with Lipofectamine 2000 (Invitrogen) at a ratio of 3 μl Lipofectamine 2000 to 1.5 μg DNA according to the manufacturer's instructions. Transfection was performed at a ratio of 1 μl ExFect to 1 μg DNA using ExFect Transfection Reagent. Use 500 ng of h-Fos-C or its truncation mutant and 500 ng of f-prk-N4BP1 or 25 ng, 50 ng, 100 ng, and 200 ng of f-prk-N4BP1 with desired vector for a total of 500 ng. After 8 to 24 h of incubation, the transfection medium was replaced with a fresh medium, and the expression of genes was detected after 48 h.

### Protein extraction and Western blotting

The collected cells were placed on ice, and an appropriate amount of radioimmunoprecipitation assay buffer (R0020, Solarbio) was added. PMSF (10 μl per 1 ml radioimmunoprecipitation assay) was added to 1 mM. After lysis, the supernatant was collected by centrifugation. SDS-PAGE protein loading buffer (5×) was added and heated to 105 °C. Protein extracts were separated by electrophoresis on 6 to 15% SDS-PAGE gels (30% acrylamide, 1.5 M Tris–HCl, 10% SDS, 10% ammonium persulfate, and tetramethylethylenediamine) and then transferred to polyvinylidene difluoride membranes. The polyvinylidene difluoride membrane was blocked in Tris buffered saline with Tween containing 5% milk for 2 h. Membranes were incubated with primary antibodies overnight at 4 °C. Antibodies for Western blotting include: anti-flag (F1804, Sigma-Aldrich), anti-GAPDH (sc-365062, Santa), anti-C-Fos (sc-166940, Santa), anti-N4BP1 (A8474, ABclonal), anti-Rent3 (sc-398821, Santa), anti-Rent1 (sc-393594, Santa), anti-Hsp70 (sc-24, Santa), and anti-CROP (14504-1-AP, Proteintech). Anti-flag, anti-C-Fos, anti-N4BP1, and anti-GAPDH were used at 1:2000 dilution for experiments, and anti-Rent3, anti-Rent1, anti-Hsp70, and anti-CROP were used at 1:1000 dilution for experiments. Membranes were then incubated with horseradish peroxidase–conjugated secondary antibody (1:2000) at 37 °C for 2 h. Signal intensity was detected with enhanced chemiluminescent substrate kit (BL520B). All samples of one experiment were treated and collected simultaneously. The loaded protein amount for each sample in one experiment was the same and it was determined by the linear and proportional response between sample loading and band intensity. The band intensity is in the linear range of chemiluminescence detection. The images shown are representative of three independent biological repeats. All images were continuously exposed to confirm the band was in the linear range of chemiluminescence detection.

### RNA isolation and quantitative real-time PCR

Total cellular RNA was extracted using the Trizol method. Two-step quantitative reverse transcription-polymerase chain reaction was used for detection. The first step uses HiScript III RT SuperMix for quantitative polymerase chain reaction (+genomic DNA wiper) reverse transcription reagent to reverse 1 ug extracted cellular RNA to synthesize complementary DNA, and the second step uses AceQ quantitative polymerase chain reaction SYBR Green Master Mix reagent to amplify DNA samples. The primers were listed at Table S1.

RT-PCR relative quantification adopts the 2-△△Ct method, which calculates gene expression differences through the difference between the Ct value of the internal reference gene. Data are presented as fold change between the treated group and the untreated group.

### Statistical analysis

The research data of this article were statistically analyzed by sigmaplot software (https://grafiti.com/sigmaplot-detail/), and all data are described in the form of mean ± SD; the unpaired two-tailed *t* test was used for comparison between groups, where ∗*p* < 0.05; ∗∗*p* < 0.01; and ∗∗∗*p* < 0.001.

## Data availability

All data generated or analyzed during this study are included in this article and the supporting information files. Any additional data and original data presented in this article are available from the corresponding author upon request.

## Supporting information

This article contains supporting information.

## Conflict of interest

The authors declare that they have no conflicts of interest with the contents of this article.
