# Supplementary material for: The NEDD4-binding protein N4BP1 degrades mRNA substrates through the coding sequence independent of nonsense-mediated decay
Source: J Biol Chem. 2024 Nov 2;300(12):107954. doi: 10.1016/j.jbc.2024.107954 (PMC11648238; doi:10.1016/j.jbc.2024.107954)
Supplement: Supplemental Tables S1–S3 [file mmc1.docx]

**The NEDD4-binding protein N4BP1 degrades mRNA substrates through the coding sequence independent of nonsense-mediated decay**

Wen Zheng ^1, 2^, Jinjing Guo ^1, 2^, Shuyan Ma ^1, 2^, Rong Sun ^2^, Yihua Song ^3^, Yuanmeng Chen ^1, 2^, Renfang Mao ^4, *^, Yihui Fan ^1, 2, *^

**Supplementary Table S1: List of RT-PCR Primers**

| Name | sense | antisense |
| --- | --- | --- |
| Human-18S | GAACGAGACTCTGGCATGCTA | CACGCTGAGCCAGTCAGTGTA |
| Human-β-Actin | TTGTTACAGGAAGTCCCTTGCC | ATGCTATCACCTCCCCTGTGTG |
| Human-JUNB | CAGGAGGGCTTCGCCGACGGC | AGTAGCTGCTGAGGTTGGTGT |
| Human-JUNC | GAACGTGACAGATGAGCAGGA | CCGGCGGCTCGCTGTGCAGGC |
| Human-FOSB | GAGGAGAAGCGAAGGGTGCGC | CCCGGTTTGTGGGCCACCAGC |
| Human-FOSC | AGCTGACTGATACACTCCAAG | CAGGCAGGTCGGTGAGCTGCC |
| Human-FOSCN | GCATGGGCTCGCCTGTCAACG | CACTGCAGGTCCGGACTGGTC |
| Human-FOSC-C | GTCAAGAGCATCAGCAGCATG | GCAGCTGGGAGTACAGGTGAC |
| Mouse-JUNB | TCACGACGACTCTTACGCAG | CCTTGAGACCCCGATAGGGA |
| Mouse-FOSB | TTTTCCCGGAGACTACGACTC | GTGATTGCGGTGACCGTTG |
| Human-N4BP1 | CCTCTGGGAAGAAGTGGACC | TTTGGCAGGGCACTGAGTAG |
|  |  |  |

| **Supplementary Table S2: List of oligo sequences for sgRNAs**   \| Name \| Oligo sequence \| \| --- \| --- \| \| SgN4BP1-1 \| GCAGAGCCGCGGCCGTATCG \| \| SgN4BP1-2 \| GGCCGTATCGAGGGCCTGTT \| \| SgN4BP1-3 \| GAGCTGCTGGAGCAGAGCCG \| \| SgUPF1-1 \| GAAAGTGAGAGTCTGCGAGC \| \| SgUPF1-2 \| GCTCGGCCTCCTCCGTGTCC \| \| SgUPF1-3 \| GACTCTCACTTTCCTGGACA \| \| SgUPF3A-1 \| GACAGCTTCTCCCTCCCGCT \| \| SgUPF3A-2 \| GCGGTGGAACTGCACTTCTA \| \| SgUPF3A-3 \| CCACCGCGACTCGCAGCAGC \| \| SgUPF3B-1 \| GTAACCCTGTTAACCCCCGC \| \| SgUPF3B-2 \| GGCCCCGGCGGGGGTTAACA \| \| SgUPF3B-3 \| GACCTCGGGGGACAGCTCCA \| \| SgLUC7L3-1 \| GTTGCTGCGCTTCTCGTCCG \| \| SgLUC7L3-2 \| GAAGCGCAGCAACGTGCGGT \| \| SgLUC7L3-3 \| GCAGTTGTTGGATGAGTTAA \| |  |
| --- | --- | --- | --- | --- | --- | --- | --- | --- | --- | --- | --- | --- | --- | --- | --- | --- | --- | --- | --- | --- | --- | --- | --- | --- | --- | --- | --- | --- | --- | --- | --- | --- | --- |

**Supplementary Table S3: List of Primers for mutants cloning**

| Name | sense | antisense |
| --- | --- | --- |
| FOS-CDS+3’UTR | CGGAATTCATGATGTTCTCGGGC | CGGGATCCCCACATGTCAAAAGACC |
| FOS-CDS | CGGAATTCATGATGTTCTCGGGC | CGGGATCCTCACAGGGCCAGCAGC |
| cFOSCDS900 | CGGAATTCATGATGTTCTCGGGC | CGGGATCCTGCATAGAAGGACCCA |
| cFOSCDS1070 | CGGAATTCATGATGTTCTCGGGC | CGGGATCCGCAGCTGCACAGCTGG |
| cFOSCDS699 | CGGAATTCGCCACCATGGATTACAAGGACGACGATGACAAGATGATGTTCTCGGGC | CGGGATCCTCACGGGGTGGCAACCTCT |
| cFOSCDS501 | CGGAATTCGCCACCATGGATTACAAGGACGACGATGACAAGATGATGTTCTCGGGC | CGGGATCCTCACGCTTGGAGTGTATCA |
| cFOSCDS300 | CGGAATTCGCCACCATGGATTACAAGGACGACGATGACAAGATGATGTTCTCGGGC | CGGGATCCTCAGGCGGGGACTCCGAAA |
| cFOSCDS225 | CGGAATTCGCCACCATGGATTACAAGGACGACGATGACAAGATGATGTTCTCGGGC | CGGGATCCTCACCACTGCAGGTCCGGAC |
| cFOS301-1143 | CGGAATTCGCCACCATGGATTACAAGGACGACGATGACAAGCCCTCCGCTGGGGCTTACT | CGGGATCCCACAGGGCCAGCAGC |
